# Polymer Binders of Ceramic Nanoparticles for Precision Casting of Nickel-Based Superalloys

**DOI:** 10.3390/nano11071714

**Published:** 2021-06-29

**Authors:** Paweł Wiśniewski

**Affiliations:** Faculty of Materials Science and Engineering, Warsaw University of Technology, Wołoska 141, 02-507 Warsaw, Poland; pawel.wisniewski@pw.edu.pl; Tel.: +48-22-234-8157

**Keywords:** binders, precision casting, ethyl silicate, hydrolysed ethyl silicate, nano-Al_2_O_3_, nano-SiO_2_, polycaprolactone (PCL), shell moulds, SiC, resins

## Abstract

This study presents the general characteristics of binders used in precision casting of Nickel-based superalloys. Three groups of binders were described: resins, organic compounds, and materials containing nanoparticles in alcohol or aqueous systems. This study also includes literature reports on materials commonly used and those recently replaced by water-soluble binders, i.e., ethyl silicate (ES) and hydrolysed ethyl silicate (HES). The appearance of new and interesting solutions containing nano-alumina is described, as well as other solutions at the initial stage of scientific research, such as those containing biopolymers, biodegradable polycaprolactone (PCL), or modified starch. Special attention is paid to four binders containing nano-SiO_2_ intended for the first layers (Ludox AM, Ludox SK) and structural layers (EHT, Remasol) of shell moulds. Their morphology, viscosity, density, reactions, and electrokinetic potential were investigated. The binders were characterized by a high solid-phase content (>28%), viscosity, and density close to that of water (1–2 mPa·s) and good electrokinetic stability. The nanoparticles contained in the binders were approximately spherically shaped with an average particle size of 16–25 nm.

## 1. Introduction

Investment casting is the primary method for manufacturing aircraft turbine parts from nickel superalloys because it can be used to fabricate products with intricate geometries.

When used for turbines, the process consists of the following steps:

(a) preparation of a wax pattern,
(b) fabrication of a multilayer ceramic shell mould on the readymade wax pattern,
(c) dewaxing/stripping,
(d) burn off of a ceramic shell mould,
(e) casting, in which ceramic shell moulds are filled with metal alloys (e.g., IN713C, IN100, Mar 247, CMXS 6, M509),
(f) hardening and hammering shell moulds,
(g) finishing and assessing the properties of the readymade shell moulds.

The process of making subsequent layers in a casting mould using the lost-wax process requires the use of appropriate binding agents to ensure the production of ceramic moulds with adequate parameters, i.e., high heat resistance, mechanical strength, gas permeability, dimensional stability, and required surface smoothness of the first layer [1,2,3,4,5,6,7,8]. The basic materials for the production of sand and ceramic casting moulds are binders, ceramic powders, and auxiliary materials, i.e., agents that limit foaming during mixing, wetting agents, pH stabilizers, and modifiers of rheological properties of the ceramic slurry [9,10,11,12,13,14]. 

Binders are liquid substances used to bind ceramic matrix particles. These are mostly synthetic materials with different chemical natures produced mainly by the chemical and food industries [15]. Binders can be divided according to the following criteria: consistency (solid or liquid); chemical nature (organic, inorganic, or organic-inorganic); type of bonding (chemical, by solidification, or by dehydration); binding capacity (low, medium, or high); and bonding temperature (sub-zero, ambient, or elevated temperatures) [16,17,18].

The binders used for the production of ceramic slurries and casting moulds determine their physical and mechanical properties. Thus this paper reviews selected binders used in the precision casting of Nickel-based superalloys. 

## 2. Synthetic Resins

Synthetic resins are amorphous high-molecular-weight products obtained by polymerization, especially by condensation. Polymerization is a chemical reaction in which low-molecular-weight compounds (monomers) are transformed into high-molecular-weight compounds (polymers). In the casting industry, intermediate products (oligomers) are mainly used, in which the final stage of the reaction takes place only in the moulding or core mass. Polycondensation, on the other hand, is a chemical reaction involving one or more types of monomers, with the chemical composition of the resulting polymer differing from that of the starting monomers. The reaction proceeds with the release of low-molecular-weight by-products. Polycondensation is a stepwise reaction, in which the monomers must have at least two reactive functional groups. Polycondensation yields phenol-formaldehyde, melamine-formaldehyde, urea-formaldehyde, aldehyde, polyester, ketone, xylene-formaldehyde, sulphonamides, and polyamide resins. The first resin used in casting was a phenol-formaldehyde resin, which was used in the Croning method. Over time, other types of synthetic resins were used, i.e., phenol-urea, urea-formaldehyde, phenol-formaldehyde-furfuryl, urea-formaldehyde-furfuryl, and acrylic resins [1,19].

Currently, new binders are being researched, including systems containing biopolymers such as BioCo polymer binder, biodegradable polycaprolactone (PCL), modified starch, methylcellulose or carboxymethyl cellulose, etc. [20,21,22]. The literature suggests it is possible to use biodegradable materials as additives in petroleum-based binders to accelerate the biodegradation of materials produced by the petrochemical industry. PCL is an example that is compatible with many other polymers. PCL is partially compatible or mechanically compatible with polymers such as polyvinyl acetate (PVAc), polystyrene (PS), polycarbonate, etc. PCL is also compatible with polymers such as polyvinyl chloride (PVC), styrene-acrylonitrile copolymer (SAN), poly(hydroxy ether), etc. [23,24,25,26]. This feature of PCL allows the creation of various blends by using it as a biodegradable component. Another benefit of using PCL as a biodegradable additive is an increase in the flexibility of the moulding sand. PCL acts as a plasticizer and also decreases the contact angle of the ceramic mass, which improves the coverage of both the wax model (in the case of the first layer) and subsequent mould layers [27,28,29,30,31,32].

## 3. Alcoholic Binders

During the precision casting of aerospace parts, the following types of alcohol-based binders are used:

- ethyl silicate (ES) and hydrolysed ethyl silicate (HES);
- silicic acid sol (SAS, e.g., Sizol 30);
- ES and HES mixtures (copolymer binders, e.g., Sikop);
- binders containing nano-silica, in which water is the diluent [19].

Until recently, binders based mostly on alcoholic (ethyl) solvents, e.g., HES, were used for the manufacture of ceramic moulds for the precision casting of turbine parts in the machine and aviation industries [33,34].

### 3.1. Ethyl Silicate

Ethyl silicate is a chemically heterogeneous mixture that is an ester of silicic acid and ethyl alcohol formed in the reaction shown in Equation (1):


SiCl_4_ + 4C_2_H_5_OH → (C_2_H_5_O)_4_Si + 4HCl (1)

It is a liquid with a characteristic odour, with a yellow or light brown colour and a density similar to water. Chemically, it is a mixture of ethyl orthosilicate, its incomplete hydrolysis products, and organic silicon compounds (e.g., a series of linear oligomers) containing ethoxy groups attached to silicon atoms. When used to make ceramic moulds, ES should contain many linear oligomers to obtain a silica content of about 40% by weight [35].

### 3.2. Hydrolysed Ethyl Silicate

Pure ethyl silicate does not show binding properties, but it undergoes efficient hydrolysis (Equation (2)):


(C_2_H_5_O)_4_Si + 4H_2_O → Si(OH)_4_ + 4C_2_H_5_OH (2)

This reaction forms water-insoluble silica that has a strong binding capacity for loose ceramic materials at elevated temperatures [36]. To initiate hydrolysis, a catalyst is needed, e.g., ethyl alcohol, acetone, hydrochloric acid, sulfuric acid, or other organic acids. The hydrolysed ES transforms into a hard, glassy, and water-insoluble material (SiO_2_) that is relatively resistant to high temperatures, is chemically inert, and can bind ceramic powders. The addition of an alcohol solvent accelerates this process but negatively affects the environment and the comfort of mould workers. 

Hydrolysed ethyl silicate retains its binding properties for 48 h after preparation. After this time, its viscosity increases due to progressive gelation. Mixtures based on alcoholic binders ensure the rapid drying of individual mould layers and the high stability of technological parameters [37,38].

By appropriately choosing reactants and water content, the volume fraction of SiO_2_ in the binder and the bond strength—which depends directly on the silicon oxide content—can be controlled. The higher the SiO_2_ concentration, the higher the bond strength and, thus, the effectiveness of HES in casting mould making [39].

### 3.3. Silicic Acid Sol

Silicic acid sol (SAS) is a suspension of silica in an aqueous medium, in which the diameter of the SiO_2_ particles is in the range of 1–100 µm. In freshly prepared SAS, the particles are highly dispersed. The binder ages over time, which is manifested as the polymerization and aggregation of silicic acid molecules. After exceeding their critical size, gelation occurs.

It should be noted that ES, HES, and SAS-type binders, due to their relatively short storage time, are often prepared at foundries. On the other hand, copolymer binders and water binders are primarily commercial products that must be purchased from dedicated suppliers [19].

### 3.4. ES and HES Mixtures

Binders in this group combine the advantages of ES and SAS. The patent [40] shows that ethyl silicate hydrolyses to form polysilicic acid, which then reacts with SiO_2_ nanoparticles. The binder contains block copolymers, which are comprised of blocks of oligomers of polysilicic acid and polyanoxysilicolates in a water-alcohol system with a pH of 1–2. Blocks of polysilicic acid molecules are formed by the acidification of a colloidal silica solution. The hydrolytic condensation of ethyl silicates is carried out in an ethanol solution. The finished binder is a copolymer with a maximum of 30% alcohol, making it non-flammable and safer to use than ES. Other features of copolymer binders are:

- long storage life and parameter stability;
- good surface wettability of the wax model;
- longer drying process of individual layers of a ceramic mould (up to 4 h), which is about 1.5 times longer than the moulding systems based on ES;
- sufficient strength of a casting mould obtained with a binder which is a mixture of ES and HES;
- ease of binder preparation during precision casting [41].

Due to increasingly strict environmental standards concerning the use of harmful and hazardous substances in technological processes, modern precision casting facilities specialising, for example, in the production of engine parts, have stopped using binders based on ethyl silicate and have switched to aqueous colloidal silica suspensions [42,43]. To improve the strength and flexibility of casting moulds, liquid polymers based on poly(vinyl alcohol) (for acidic systems) or latexes (for alkaline systems) have recently been introduced into binders. Polymer-modified binders have become the subject of patents. Today, the leading suppliers of aqueous binders and additives for moulding systems are Ransom & Randolph (USA) and REMET (UK).

## 4. Water Binders

### 4.1. Water-Soluble Binder Containing Nano-Al_2_O_3_

Several other binders are used in the manufacture of ceramic casting moulds, but their use is limited for various reasons. An example is the Imerys binder containing nano-Al_2_O_3_ (Evonik, Germany), which has been applied in creating Y_2_O_3_ moulding sands intended for casting mould relief layers, moulding sands, and moulds [44,45,46,47,48,49,50].

Table 1 shows the basic properties of the used casting binder containing nano-alumina.

**Figure 1:**
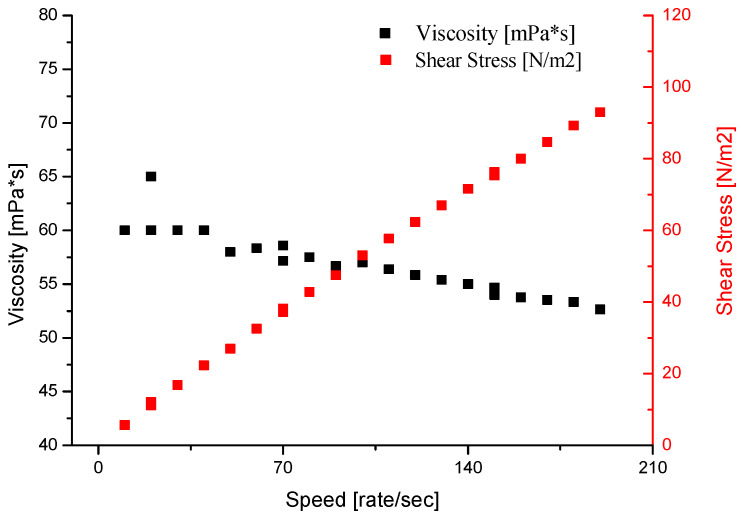
Dynamic viscosity versus shear rate (black curve) and shear stress versus shear rate (red curve) of Imerys W440 binder.

**Figure 2:**
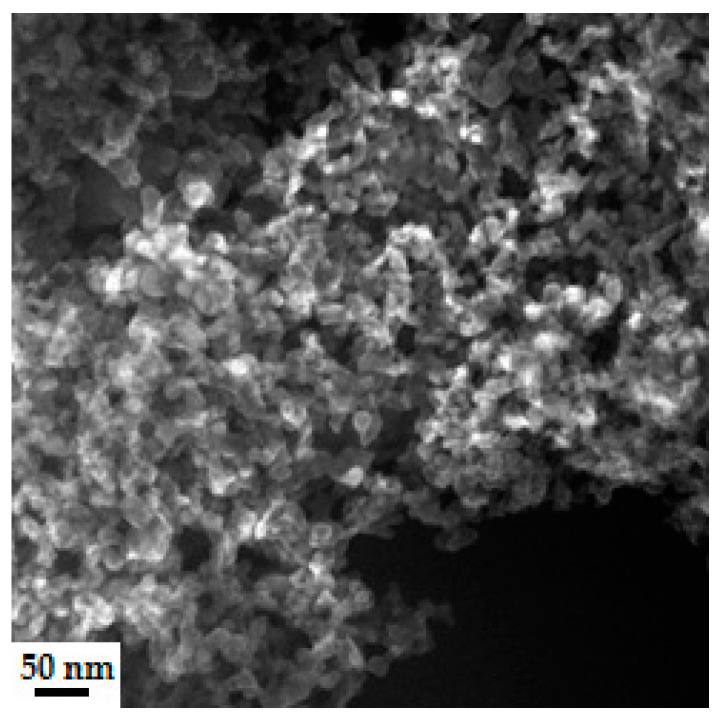
Morphology of Al_2_O_3_ nanoparticles in Imeris W440 polymer binder.

The binder density was determined using a glass areometer with a measuring range up to 1.5 g/cm^3^. The pH of the binder was measured with a sensION-1 (Hach, Vienna) pH meter, equipped with an electrode for suspensions. Figure 1 shows the dynamic viscosity result of the W440 binder, and Figure 2 shows an example of the morphology of Al_2_O_3_ nanoparticles in the binder. 

The dynamic viscosity testing of the binder was performed in a TG Instruments Ares 4400 analyser (USA) in a plate–plate configuration at increasing shear rates in the range of 5–200 s^−1^. The analysed fluid was characterized by pseudoplastic properties, i.e., its viscosity decreased at increasing shear rates from 61 to 54 mPa·s (Figure 1).

Figure 2 shows an example of the morphology of a dried binder on a graphite grid. The study was carried out using a Hitachi 5500 SEM/TEM (Tokyo, Japan) using an accelerating voltage of 300 kV. The binder was diluted 10 times and applied to copper grids and then sputtered with osmium. It can be seen from the images that the Al_2_O_3_ nanoparticles contained in the binder are globular and equiaxial in shape. The polymer component of the binder coated the surface of the alumina particles and caused them to strongly agglomerate. Based on stereological measurements, the average particle size of nano-Al_2_O_3_ was estimated to be 16 nm [45].

**Figure 3:**
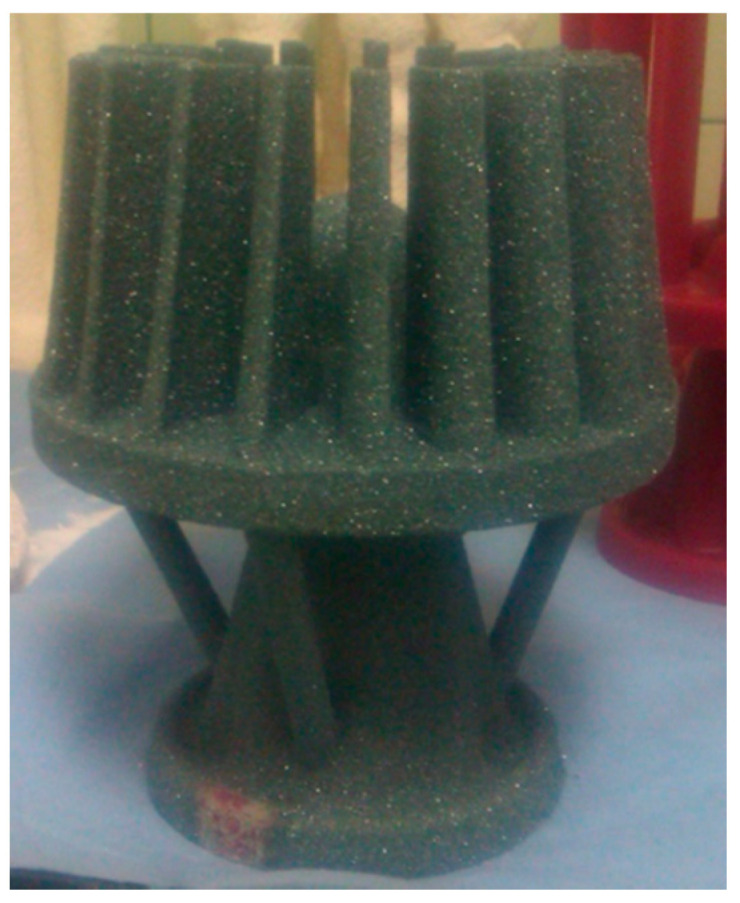
Example SiC mould (raw state) obtained using Imerys W440 binder.

Figure 3 shows an example of a SiC mould in its raw state (after the 3rd layer was applied) obtained using the Imerys W440 binder. The suitability of this binder for obtaining actual silicon carbide casting moulds was confirmed.

### 4.2. Water-Soluble Binders Containing Nano-SiO_2_

Colloidal silica is a hydrosol of orthosilicic acid dispersed in water. Orthosilicic acid is obtained by acidifying a silicate solution (e.g., Na_2_SiO_3_ or water glass). The resulting silicic acid sol, due to water binding, spontaneously dehydrates to SiO_2_ through di-, tri-, etc. silicic acid steps, followed by polysilicic acid steps (with decreasing amounts of water). The final result is a colloidal silica sol dispersed in water. The SiO_2_ particles in the colloidal solution are spherical and range in size from a few to tens of nanometers [51]. 

Among the competing green materials, binders based on colloidal silica (CS), a hydrosol of silicic acid suspended in water, are the most commonly used binders in the casting industry. The binding agent for binding the ceramic powder particles is silica gel, which is formed when water is evaporated from the sol. Compared with alcohol-based binders, colloidal silica, is environmentally neutral and harmless to workers [52].

Compared with alcohol-based binders, water-based binders also have several disadvantages. The main ones include longer drying and curing times of individual mould layers and their instability over time, which makes it necessary to check the properties of ceramic mixtures and casting moulds [53]. 

**Figure 4:**
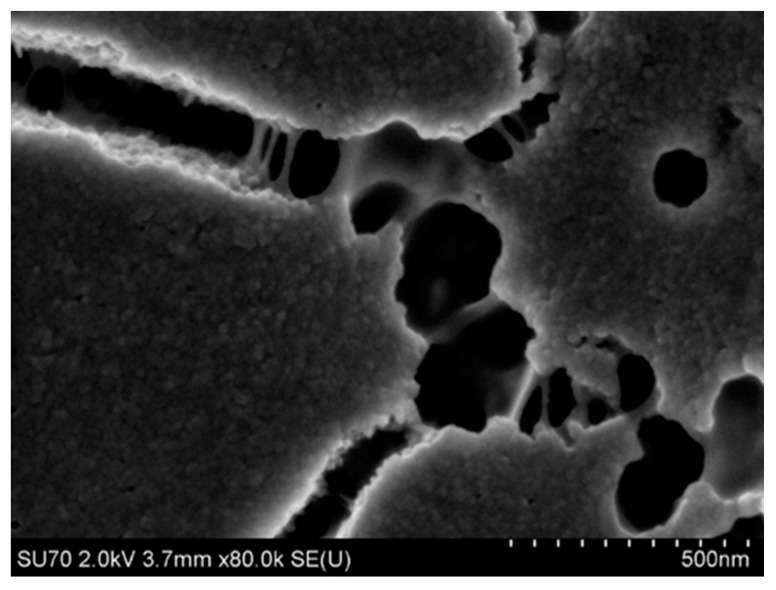
Example morphology of a mixture of the Remasol-aluminosilicate system after gelation.

In the first densification step, a colloidal solution of 3–5% SiO_2_ up to a concentration of 25–50% was obtained. Before or during the thickening process, the diluted colloidal silica is subjected to a stabilization and modification process. For this purpose, e.g., sodium, alginate, or aluminium salts, and polymers such as latex or poly(vinyl alcohol) are introduced to maintain an appropriate charge on the surface of SiO_2_ nanoparticles and to provide appropriate strength and “elasticity” of the binder after gelation, i.e., in the hardened state [54]. An example of the microstructure of the Remasol binder in the cured state is shown in Figure 4. Polymer “bridges” can be seen, which ensure cohesion, despite cracks in the layer. 

**Figure 5:**
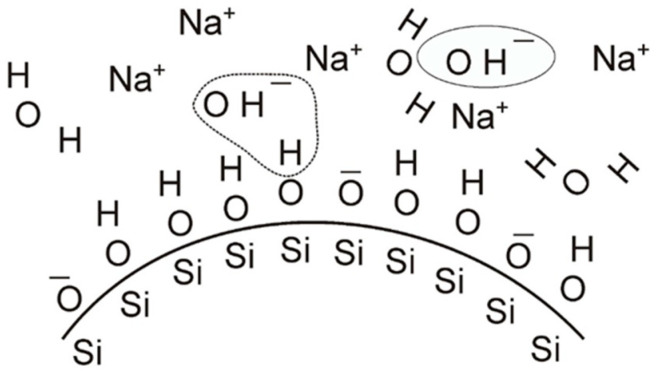
Schematic of the charge formation on the surface of SiO_2_ particle in colloidal silica-based binder stabilized by sodium hydroxide, adapted from [53].

As a standard, binder stability is ensured by maintaining an appropriate pH (usually strongly alkaline) by introducing sodium or ammonium hydroxide into the solution. As shown in Figure 5, OH^-^ ions react with the H^+^ ion of the hydroxyl group from the surface of the SiO_2_ particles. The reaction produces a water molecule, and an unbalanced negative charge appears on the surface of the SiO_2_ particle. The negatively-charged SiO_2_ particles repel each other so that they remain uniformly dispersed in the dispersing medium without agglomeration. A major inconvenience with this stabilization is that the particle charge strongly depends on the pH. Unavoidable changes in pH during mix preparation and use may result in accelerated ageing of the binder or premature gelation. The pH should therefore be continuously monitored and maintained at an appropriate level [55].

**Figure 6:**
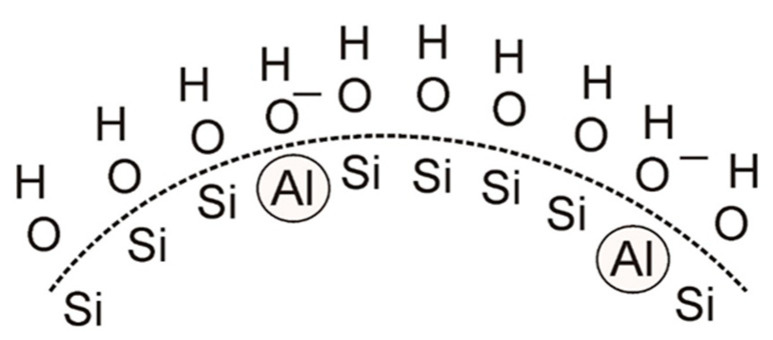
Charge formation on the surface of SiO_2_ particles in the binder stabilized with an aluminium salt, adapted from [53].

There are other ways to modify binders with water-soluble aluminium salts. The incorporation of Al^3+^ cations into the silicon chain (Figure 6) results in an unbalanced negative charge on the surface of the SiO_2_ particle regardless of the pH of the surrounding environment. The appearance of this charge is the result of the different valences of silicon and aluminium cations. 

**Figure 7:**
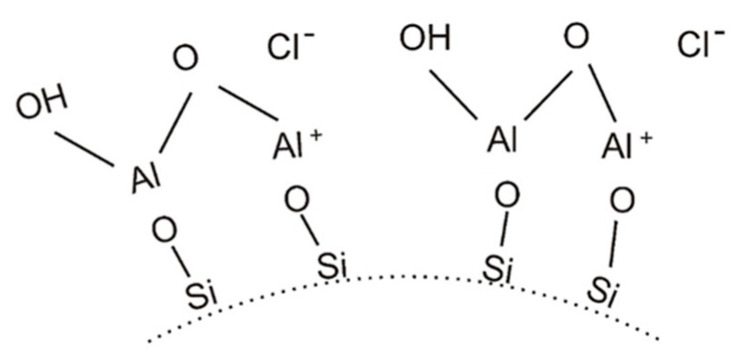
Surface modification of a SiO_2_ particle to change the particle charge to positive, adapted from [53].

It is also possible to modify (coat) the surface of SiO_2_ particles in the colloid with hydrated aluminium oxide, which forms a positive charge on the surface of the colloidal particles (Figure 7).

**Figure 8:**
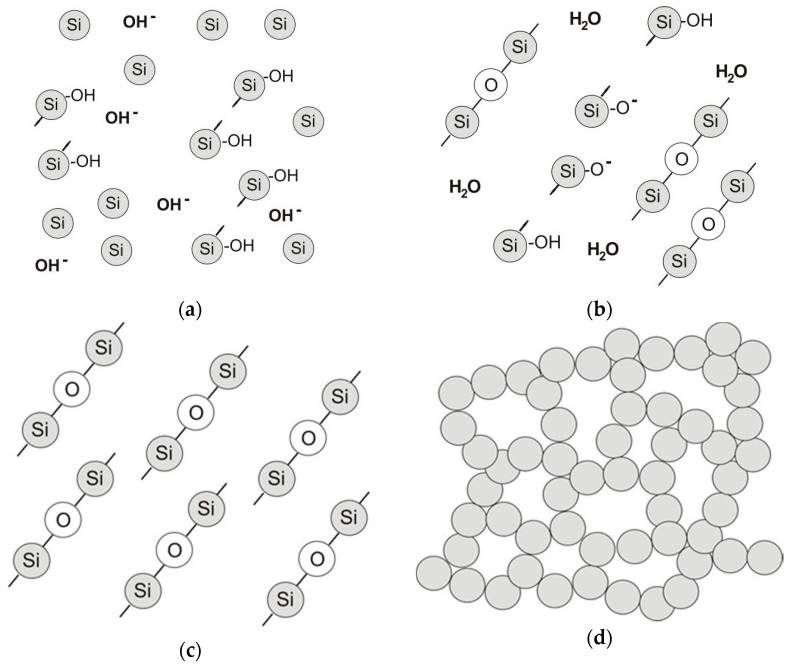
Binding scheme of colloidal silica-based binders: (**a**) chemical structure of the colloidal system, (**b**) reaction between hydroxyl groups from the surface of SiO_2_ particles leading to the creation of siloxane bonds, (**c**,**d**) three-dimensional chains of SiO_2_ particles. Adapted from [53].

The technological properties of binders based on colloidal silica are influenced by the content and size of SiO_2_ particles, chemical composition (presence of stabilizers and modifiers), reaction solution, time, and temperature. Colloidal silica is environmentally benign [55]. The binding of the colloidal silica-based binder is related to the sol-gelation process, which is accomplished via water evaporation. During drying, due to the reaction between hydroxyl groups on the surface of silica particles, SiO_2_ particles irreversibly combine into spatial structures with -Si-O-Si- bonds, as shown in Figure 8 [56]. 

The advantages of colloidal silica-based binders include [57]:

(a) long lifetime (period of technological usefulness),
(b) good surface wettability of the wax model,
(c) sufficient strength of the mould throughout the whole technological process,
(d) ease of binder preparation in facility conditions.

An undoubted disadvantage of these binders is the relatively long drying and hardening time of subsequent ceramic layers applied on the wax model compared with the time required to harden the layers formed using ethyl silicate.

### 4.3. Properties of Selected Binders Based on Colloidal Silica

The basic technological properties that should be determined for colloidal silica-based binders before preparing the ceramic mass used in fused model technology are [58]:

- content of solid phase in binders,
- wettability of binders,
- viscosity and density of binders,
- bonding capacity of binders.

The following section presents examples of the properties of two commercially-available binders used to produce the first mould layer, Ludox AM and Ludox SK (Remet UK), and two other binders used to create structural layers of the ceramic moulds, EHT (Ransom and Randolph USA) and Remasol (Remet UK).

#### 4.3.1. External Appearance, pH, and Solid-Phase Content of Selected Binders Based on Colloidal Silica

**Figure 9:**
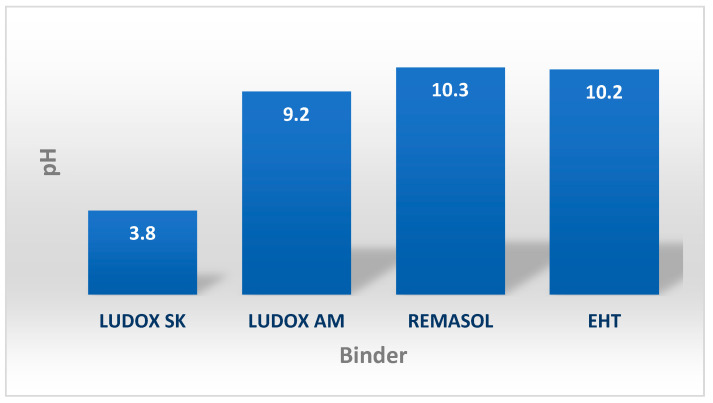
The reaction of selected binders based on colloidal silica.

The simplest way to evaluate silica-based binders is to evaluate their colour, homogeneity (delamination, deposits, and impurities) and check their odour. When properly stored and handled, a nano-SiO_2_-based binder should be a clear solution with a uniform colour throughout. Binders may be completely transparent (Ludox AM) or milky white (EHT) and odourless (Ludox SK) or with strong irritating odours (Remasol). Another test to confirm the usability of a binder is to meaure the reaction’s progress. Example results of pH measurements for selected binders are shown in Figure 9.

As a rule, binders based on colloidal silica undergo an alkaline reaction; however, acidic binders are also available, such as Ludox SK. The reaction of the binder depends on the method of stabilization and the type of water-soluble polymers added. The nature of the reaction of the binder determines the type of matrix (ceramic dust) from which the ceramic mass will be prepared. The ceramic dust (matrix) should not significantly change the pH of the prepared liquid ceramic mass, as this may decrease the stability of the colloidal system because it may fall into an unstable state. 

**Figure 10:**
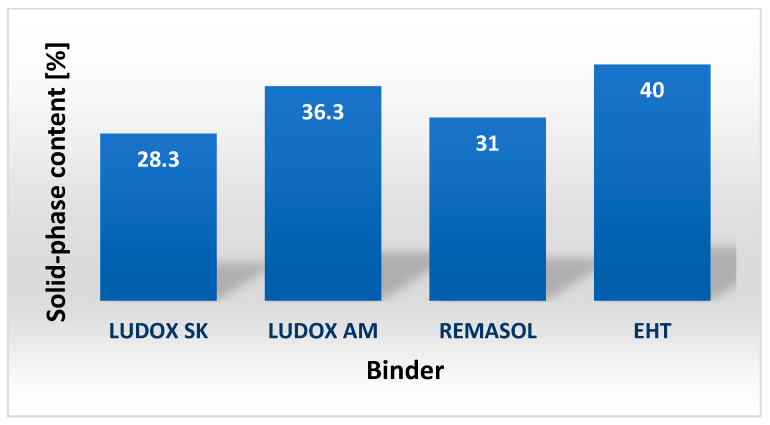
Solid-phase content of selected binders based on colloidal silica.

The solid-phase content and wettability of hydrophobic surfaces are important technological parameters for characterizing binders. The solid-phase content is determined by measuring the weight of a binder before and after water evaporation. Figure 10 shows the measured solid contents of colloidal silica-based binders. In the precision casting of nickel and cobalt alloys, binders with a solid-phase content between 25 and 40% by weight are used. From the obtained results, it can be seen that the proportion of solid phase in all these binders is within this range.

#### 4.3.2. Microstructure of Polymeric Binders Based on Colloidal Silica

**Figure 11:**
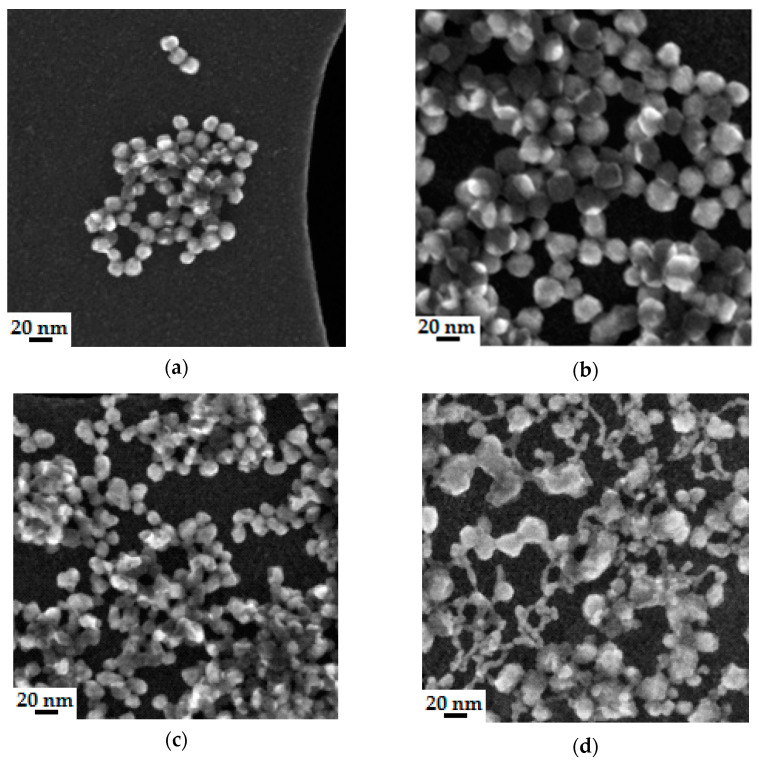
Morphology of nano-SiO_2_ in the binders: (**a**) LUDOX SK, (**b**) LUDOX AM, (**c**) REMASOL, (**d**) EHT.

Figure 11 shows the morphology of SiO_2_ nanoparticles in colloidal silica-based polymer binders.

As shown in Figure 11, SiO_2_ nanoparticles contained in the tested binders were spherical, similar to the Imerys W440 binder. This is especially true for silica nanoparticles in Ludox SK, Ludox AM, and Remasol binders. The most irregular-shaped colloidal SiO_2_ were observed in the composition containing the EHT binder. Compared with the other binders, the silica nanoparticles in the EHT binder showed the highest agglomeration tendency (Figure 11d). Based on stereological measurements, the average particle size range of nano-SiO_2_ in the binders was estimated to be 18–20 nm.

#### 4.3.3. Zeta Potential of Selected Binders Based on Colloidal Silica

Colloidal silica-based binders have an ionic nature because there are unbalanced electric charges on the surface of SiO_2_ particles. If the amounts of positive and negative charges on the silicon oxide surface are equal, the system is in isoelectric equilibrium (IEP - isoelectric point). The zeta potential is then equal to zero, which means that at this point, the dispersion has no electrostatic stability. Since the concentration of H^+^ and OH^-^ ions depends on the pH of the solution, the isoelectric equilibrium corresponds strictly to the pH value. 

In the isoelectric state, SiO_2_ nanoparticles readily assemble into larger units, which consequently leads to agglomeration, which is driven by the attraction of dissimilar electric charges. When the zeta potential is strongly positive or strongly negative, positive or negative charges prevail on the particle surface, respectively. The unipolar charges repel each other, which hinders agglomeration, and the system is in a steady state [58].

The zeta potential, or electrokinetic potential, is defined as the difference in electrical potentials at the surface of an electrically-charged solid-phase particle. The value of this potential depends on the type of solid and the composition of the solution. The type and concentration of ions and surfactant present in the solution also affect the zeta potential value. Plotting the zeta potential curve as a function of pH allows the determination of stable and unstable areas for the binders.

In a more acidic environment, the total electrostatic external charges of the grains will be positive. In a more alkaline environment, the total surface charges will be negative. It is possible to distinguish a suspension’s stability range using the zeta potential value, as shown in Table 2. It is assumed that a stable water dispersion has a zeta potential greater than 30 mV [16].

**Figure 12:**
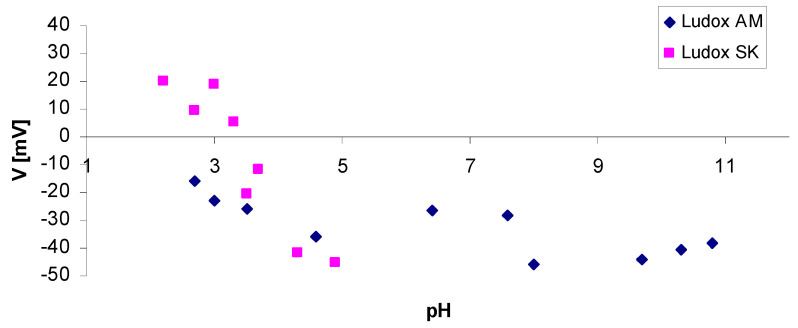
Dependence of the zeta potential on pH for Ludox SK and Ludox AM binders used to produce the first layer of a ceramic mould.

**Figure 13:**
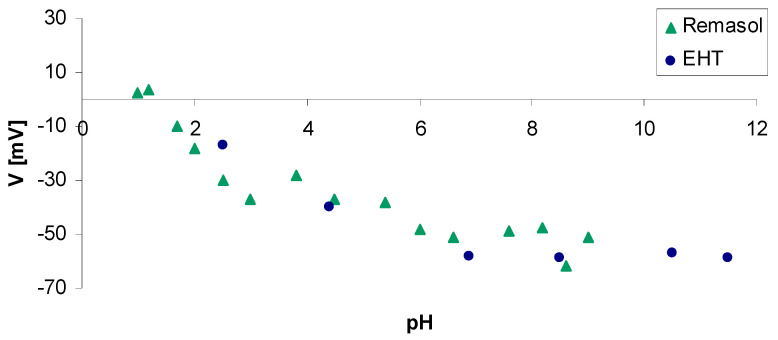
Dependence of zeta potential on pH for Remasol and EHT binders used to produce structural layers of a ceramic casting mould.

In addition to reactions, the zeta potential value is affected by changes in conductivity and changes in additive concentration, but pH has the greatest effect. Figure 12 and Figure 13 show the zeta potential changes of the binders used to prepare ceramic masses applied to model and structural layers. Measurements were performed using a ZetaSizer Nano ZS potential analyser (Malvern UK).

For Remasol, EHT, and Ludox AM binders, the zeta potential curves are similar. Isoelectric equilibrium occurs at pH = 1–2. Above this range, the zeta potential is negative over the entire measurement range, which means that the binders are stable from pH = 5.0–12.0. 

A different zeta potential curve was recorded for the Ludox SK binder. It has been shown that isoelectric equilibrium is reached at pH 3.5. Below pH 3.5, the zeta potential is positive and at pH 2.2, it reaches 20 mV. At pH values above 3.5, the potential is strongly negative and takes a value of −45 mV for pH 5.0. The presence of aluminate salt and poly(vinyl alcohol) in the Ludox SK binder significantly changed the nature of the zeta potential curve. The binder is stable in an acidic environment.

#### 4.3.4. Viscosity and Density of Selected Binders Based on Colloidal Silica

**Figure 14:**
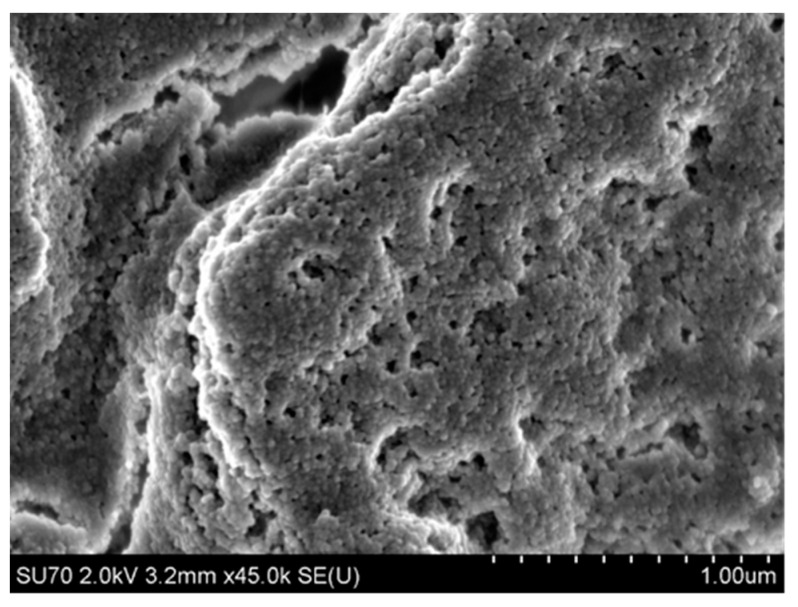
ZrO_2_ powder grain surrounded by silica nanoparticles from the Ludox SK binder.

**Figure 15:**
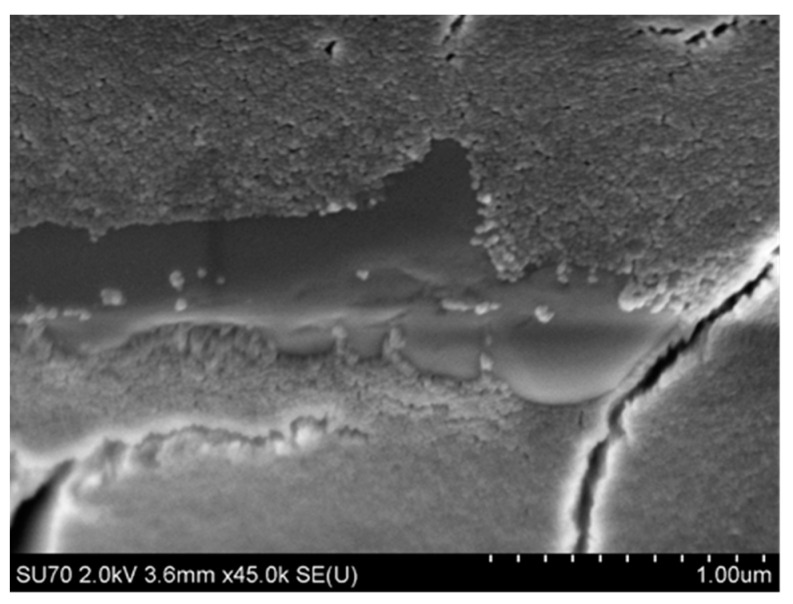
Morphology of the dried mixture of the Ludox AM-Al_2_O_3_ system.

The dynamic and kinematic viscosity and density of binders are the basic parameters for characterizing a binder in terms of its ability to cover the surface of ceramic matrix particles that form the moulding sand. Binders should have the lowest possible viscosity, which, along with good wettability, facilitates dispersion of matrix powder particles in a ceramic cast and helps surround these particles with the binder layer. Example morphologies are shown in Figure 14 and Figure 15. The surface morphology of the first dried layer in Ludox SK–ZrO_2_ powder and Ludox AM–Al_2_O_3_ powder mixed systems was revealed using a Hitachi SU-70 high-resolution scanning electron microscope (Hitachi, Tokyo, Japan) using a 2 kV accelerating voltage and a secondary electron detector. The collected mixture samples were dried at room temperature and then sputtered with osmium. 

The Ludox SK–ZrO_2_ system formed a porous layer with randomly distributed pores (Figure 14). In contrast, the Ludox AM–Al_2_O_3_ system was cracked (Figure 15), which was practically not observed in the Ludox SK– ZrO_2_ system. For both binders, the micro-grains of the moulding powders were relatively well-surrounded by the SiO_2_ nanoparticles of the binder, indicating the good wettability of both systems.

**Figure 16:**
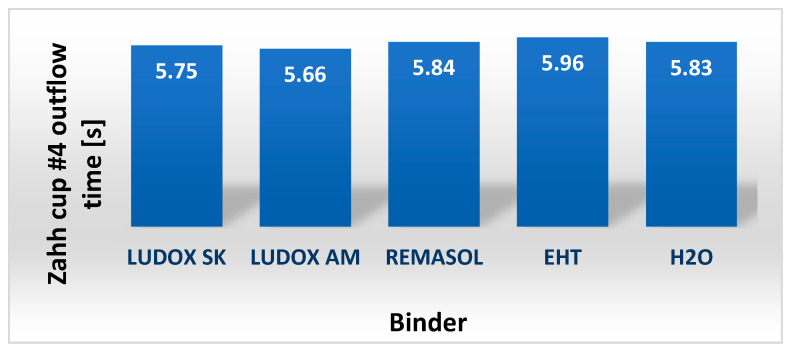
The relative viscosity of the binders determined with a Zahn cup, *T* = 21 °C.

In industrial practice, binder and moulding sand viscosity tests are carried out using Ford or Zahn flow cups. Example results of measurements of the conventional viscosity of the tested binders using a Zahn cup are shown in Figure 16. As can be seen from the cited data, currently available binders based on colloidal silica have a low viscosity, comparable to that of water.

**Figure 17:**
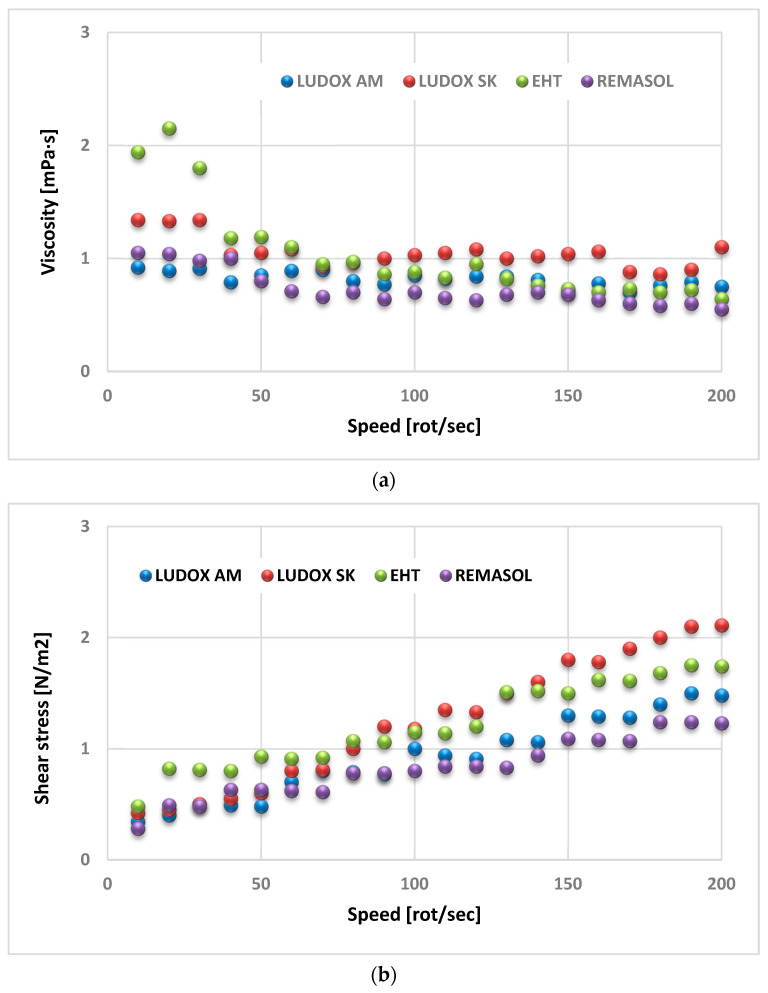
Dependence of (**a**) dynamic viscosity and (**b**) shear stress on shear rate for colloidal silica-based binders.

Methods for measuring the relative and conventional viscosity (Zahn cup flow time) are simple techniques readily used in industrial practice, but they do not allow for the complete characterization of the rheological properties of the binder; therefore, rheometers that provide quantitative dynamic viscosity and shear stress values as a function of shear rate are used increasingly frequently to evaluate the viscosity of binders. Example results of dynamic viscosity and shear stress (determined using a rotational viscometer) for the binders used to produce the first layer and the binder for the so-called mould construction layers are shown in Figure 17.

The binders used for the first layers (Ludox SK and AM) are Newtonian liquids, i.e., their viscosity does not depend on the shear rate. Ludox SK obtained an average value of 1.12 mPa·s, while for Ludox AM it was 0.8 mPa·s; therefore, the flow curves, which show the dependence of shear stress on shear rate, are straight lines described by Newton’s equation; however, the binders used for the structural layers (Remasol and EHT) are pseudoplastic liquids whose viscosity decreased upon increasing the shear rate and are not thixotropic. 

**Figure 18:**
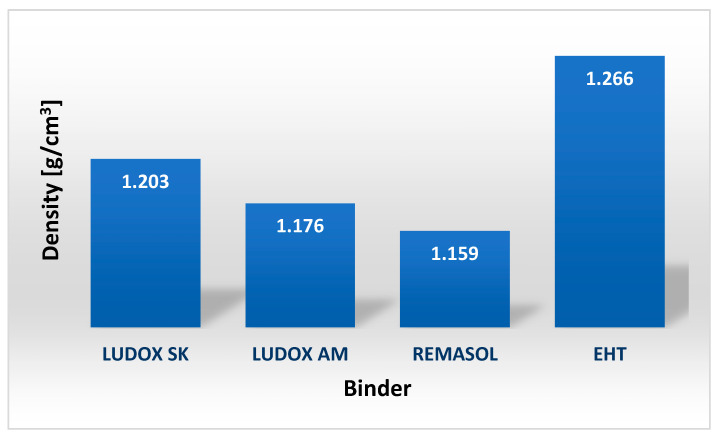
The density of binders produced based on colloidal silica.

The EHT binder has a slightly higher viscosity than the Remasol binder over the entire shear rate range. The viscosity of the EHT binder varies from 2.1 to 0.7 mPa·s and that of the Remasol binder from 1.3 to 0.6 mPa·s. The density of binders is best determined using aerometers. Figure 18 shows the densities of four selected colloidal silica-based binders, which have similar values that are only slightly higher than that of water [26]. 

**Figure 19:**
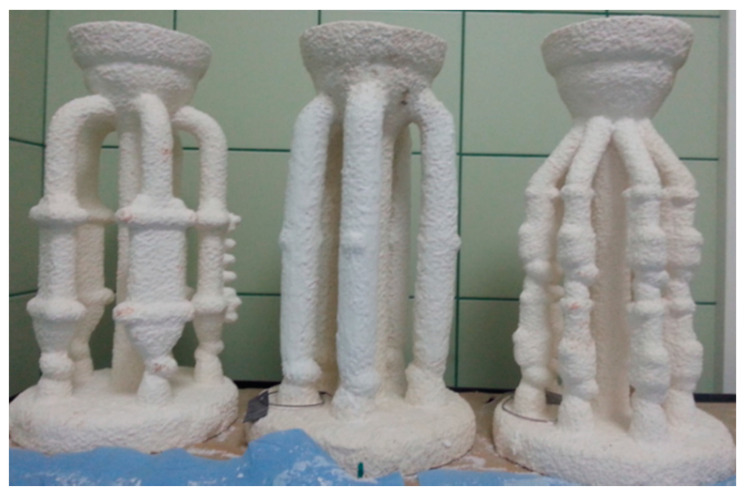
Examples of corundum ceramic moulds obtained with Ludox AM and Remasol binders.

**Figure 20:**
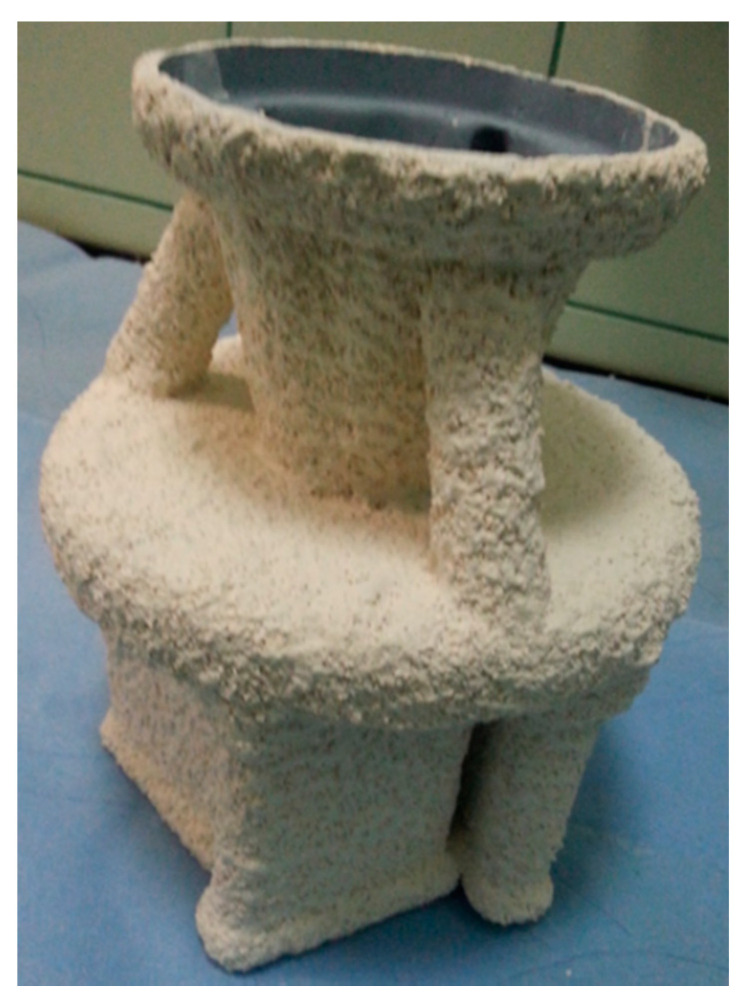
Example of a ZrO_2_ ceramic mould obtained with Ludox SK and EHT binders.

Due to their favourable properties, four selected binders were used to obtain ceramic casting moulds. Figure 19 and Figure 20 show examples of the ceramic moulds obtained with binders containing colloidal silica.

## 5. Conclusions

Binders are one of the main components of ceramic moulds that combine powder particles and provide appropriate rheological and technological properties to moulding sand and casting moulds. Until recently, alcohol (ethanol) binders based on hydrolysed ethyl silicate were used to make ceramic moulds.

Due to ecological aspects, binders based on colloidal silica are currently used, which, compared with ethylene binders, are characterized by longer drying times and a greater tendency to crack the ceramic shell in the unburnt form; however, they are characterized by good wettability and a longer technological shelf life. They also increase the mechanical strength and gas permeability of moulds compared with analogous systems based on HES. Their undoubted advantage is to ensure the comfort and safety of workers in casting facilities.

This article reviewed selected polymeric binders dedicated to precision casting of nickel-based superalloys. The most attention was paid to four binders containing nano-SiO_2_ for model layers of casting moulds (Ludox AM, Ludox SK) and for structural layers (EHT, Remasol). The binders were characterized by high solid-phase contents (28–40%), viscosities, and densities close to that of water (1–2 mPa·s; Zahn cup flow time 5–6 s), as well as good electrokinetic stability. Nanoparticles contained in the binders were characterized by a near-spherical shape and an average particle size of 16–20 nm. 

Such materials are commonly used to make casting moulds of electrocorundum, ZrO_2_, quartz, and aluminosilicates. As an alternative, nano-Al_2_O_3_ based binders can be used to make Y_2_O_3_ model layers, silicon carbide moulding compounds, and moulds. Interesting solutions in this field of research are the application of modified starch, polycaprolactone, and other polymers of natural origin.

## Figures and Tables

**Table 1:** Basic properties of binder containing nano-alumina.

Binder	Solid-Phase Content [%]	Density [g/cm^3^]	pH	Time out of Zahn’s Cup #4 [s]
Imerys W440	44.00	1.28	7.67	6.8

**Table 2:** Stability of suspension depending on the zeta potential, ξ.

Zeta Potential ξ [mV]	Suspension Properties
approx. 0–5	Fast coagulation
approx. 5–10	Slow coagulation
approx. 10–30	Instability
approx. 30–40	Variable stability
approx. 40–60	Good stability
>60	Very good stability
